# Transcatheter aortic valve implantation in elderly patients with severe aortic stenosis and rheumatic phenotype

**DOI:** 10.1007/s12928-025-01113-w

**Published:** 2025-03-31

**Authors:** Ahmed Abdelrahman Elkaialy, Nabil Farag, Ahmad Elsayed Mostafa, Mahmoud Baraka, Diaa Kamal

**Affiliations:** Department of Cardiology, Faculty of Medicine, Ain Shams University, Cairo, 11591 Egypt

**Keywords:** Transcatheter aortic valve implantation, Rheumatic, Aortic stenosis

## Abstract

Significant fibrosis is characteristic of rheumatic aortic valve disease that affects anchoring and deployment of the trans-catheter heart valve. Our goal was to determine whether trans-catheter aortic valve implantation (TAVI) is an effective treatment option for severe aortic stenosis patients with rheumatic valvular disease. This study is a prospective cohort study that includes a total of 54 rheumatic severe aortic stenosis patients who were referred for TAVI. The age ranged from 65.00 to 83.00 years (mean age 72.75 ± 5.86 years). Notably, 63% of the patients had undergone preimplantation balloon dilatation. The outcomes included total new conduction disturbances (transient and permanent defects of atrio-ventricular block and left bundle branch block) in 31.4% of the patients, permanent pacemaker implantation in 3.7%, and valve embolization in 1.85%, and no patients had moderate to severe para-valvular leak. All-cause death within a 6-month follow-up was 3.7%. This cohort was then compared to a propensity score matched group of degenerative calcific severe aortic stenosis of 54 cases revealing comparable post-procedural results. Compared with the outcomes of TAVI in patients with degenerative aortic stenosis, TAVI represents a viable and durable option for rheumatic severe aortic stenosis patients.

**Graphical abstract:**

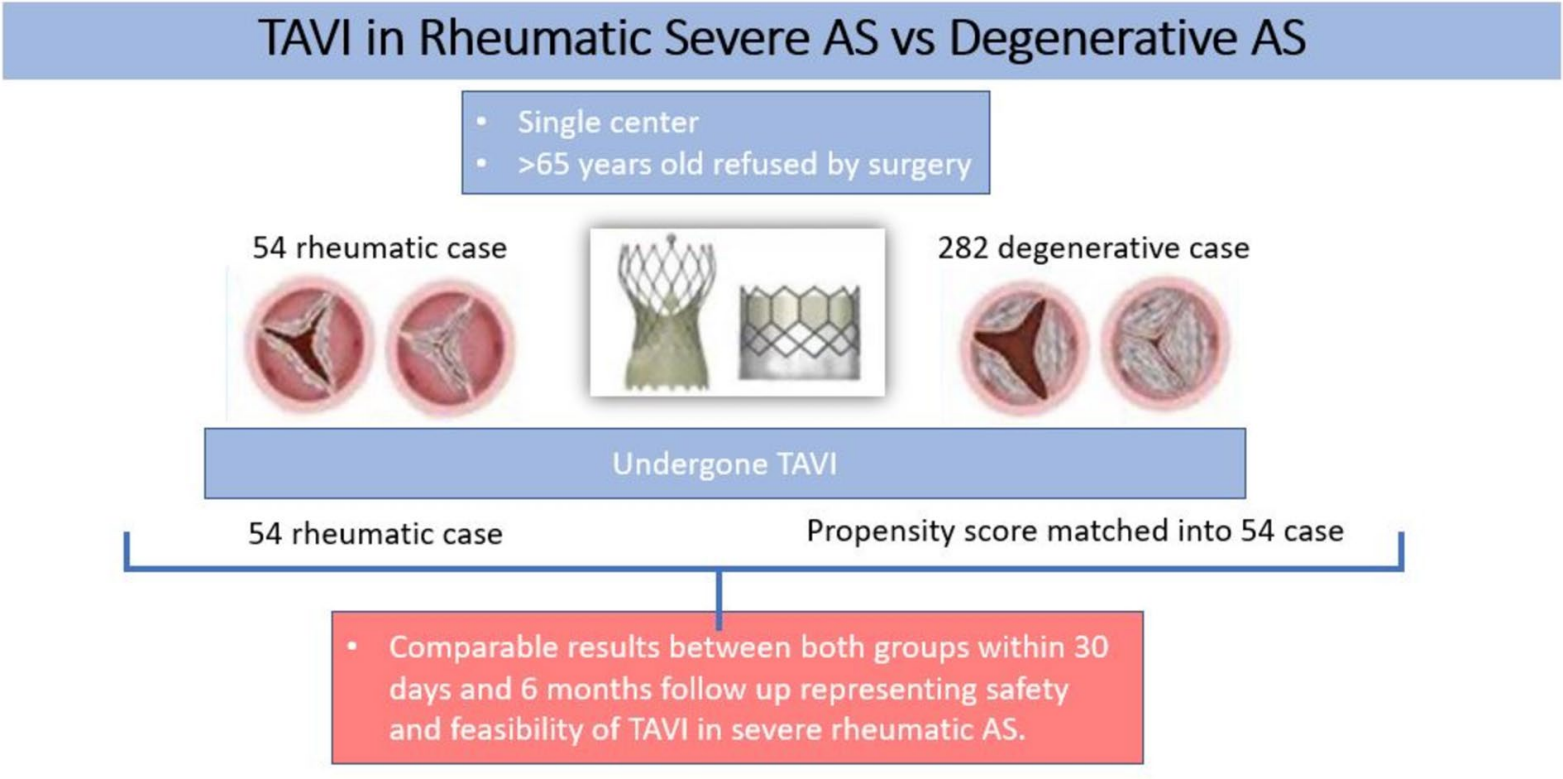

## Introduction

Significant fibrosis and late-stage calcification of the degenerative process are features of rheumatic aortic valve disease. Anatomical differences may affect the trans-catheter heart valve’s (THV) deployment and anchoring when compared to degenerative aortic stenosis (AS). The technical aspects of treating rheumatic aortic stenosis vary. In fact, commissural fusion and leaflet thickening serve as an elastic band that tends to squeeze the implanted THV resulting in a high probability of migration or embolization. Therefore, intentions to almost always pre-dilate the rheumatic aortic valve are increasing to disrupt the commissural fusion and the elastic band increasing the stability of the implanted trans-catheter heart valve. Moreover, owing to decreased leaflet calcification and lower possibility of device anchoring, the decision of greater degree of over-sizing reaching 20% becomes essential along with deeper implantation of the THV to ensure adequate anchoring of the prosthesis.

Our objective was to demonstrate that TAVI is a practical and efficient treatment option for patients with rheumatic valvular disease who have severe aortic stenosis.

## Methods

The research was carried out between January 2022 and January 2024 at the Cardiology Department of Ain Shams University Hospitals via prospective observational methods. Our registry included 336 patients of which fifty-four patients had severe rheumatic aortic stenosis. Patients were allocated into two groups; the first one included 54 rheumatic severe aortic stenosis patients and the other arm included 282 degenerative calcific severe aortic stenosis who were then propensity score matched into 54 cases. During the specified period, we performed TAVI in 54 cases representing 16% of the total 336 TAVI cases including both rheumatic and degenerative calcific pathologies. A convenience sampling approach was employed to find study participants. Patient outcomes were documented via the Valve Academic Research Consortium-3 criteria (VARC-3 criteria) [1], and patients were followed up for six months.

The inclusion criteria [according to the latest ESC guidelines of valvular heart disease (VHD)] [2] were patients above 65 years of age, fulfilling the criteria for rheumatic aortic valve by echocardiography according to the 2012 World Health Federation (WHF) criteria [3], concomitant rheumatic mitral valve affection, history of rheumatic activity, refusal by surgeons, symptomatic severe aortic stenosis (NYHA functional class, CCS grading, and syncope), aortic valve area < 1 cm^2^ or < 0.6 cm^2^/m^2^, high gradient AS (mean gradient > 40 mmHg or jet velocity > 4.0 m/s), low flow low gradient AS with impaired functions, low flow low gradient AS with preserved functions, and aortic valve annulus diameter ≥ 18 and ≤ 30 mm.

This study was authorized by the ethical committee of Ain Shams University, FMASU MD 127/2022. Each patient in the cardiology department at Ain Shams University hospitals received a thorough explanation of the process and gave their consent in a private setting. The primary researcher was the only one with access to the patient’s medical records, and the study was conducted in total secrecy. The participants were free to quit from activity at any time, and participation was entirely optional.

### Preprocedural data analysis

A diversified cardiac team comprising interventional cardiologists, cardiac surgeons, and imaging experts assessed the patients and categorized them into high, intermediate, or low risk groups. To estimate the risk of death for each patient, the European System for Cardiac Operative Risk Evaluation (EuroSCORE) II [4] and the Society of Thoracic Surgeons (STS) risk score [5] were calculated. A detailed preinterventional assessment was conducted on every instance. Every patient had an electrocardiogram, with the fundamental rhythm, axis deviation, PR interval, QRS morphology, and QRS duration recorded.

Patients had standard 2-dimensional B-mode and Doppler transthoracic echocardiography (TTE) prior to the procedure, and standard parameters were measured in compliance with guidelines provided by the American Society of Echocardiography [6].

**Fig. 1:**
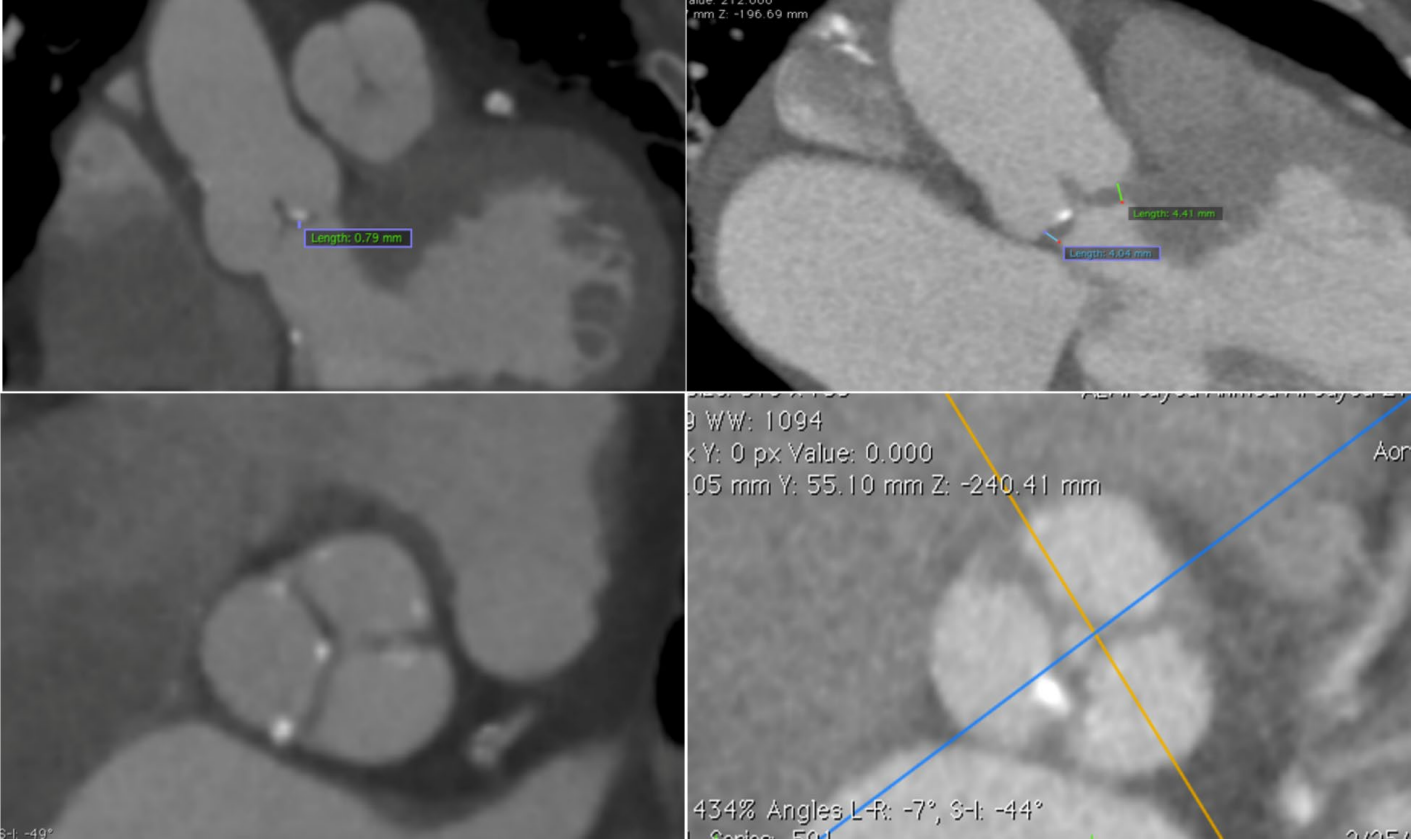
Pre-TAVI CT assessment of rheumatic severe AS

The Osirix MD version software was utilized to process all computed tomography (CT) images. The measurements included the length of the membranous septum (MS) with values indexed to BSA (MSi), the diameter of the sinus of Valsalva, the height of the left and right coronary ostia (LMCA and RCA, respectively), and the calcification degree of the valve and basal septal calcification (Fig. 1) Moreover, thickness, commissure fusion, and calcification distribution were examined and verified using two- and three-dimensional computed tomography. Aortic valve calcification grading: There was no calcification in Grade 1, moderate calcification in Grade 2 (tiny, isolated areas), moderate calcification in Grade 3 (many, larger spots), and heavy calcification in Grade 4 (extensive calcification over the entire circumference) [7].

Rheumatic mitral valve involved morphological features: (A) Commissural fusion; (B) thickening of the leaflets with or without calcification; (C) restricted mobility of the leaflets, which causes the anterior mitral leaflet to resemble a “hockey stick” or “doming” during diastole; and (D) thickening and shortening of the chords.

Rheumatic aortic valve involved morphological features: (A) Retraction of the leaflet margins; (B) fibrotic thickening; (C) commissural fusion; and (D) a triangular or rounded opening in systole.

### Procedure

**Fig. 2:**
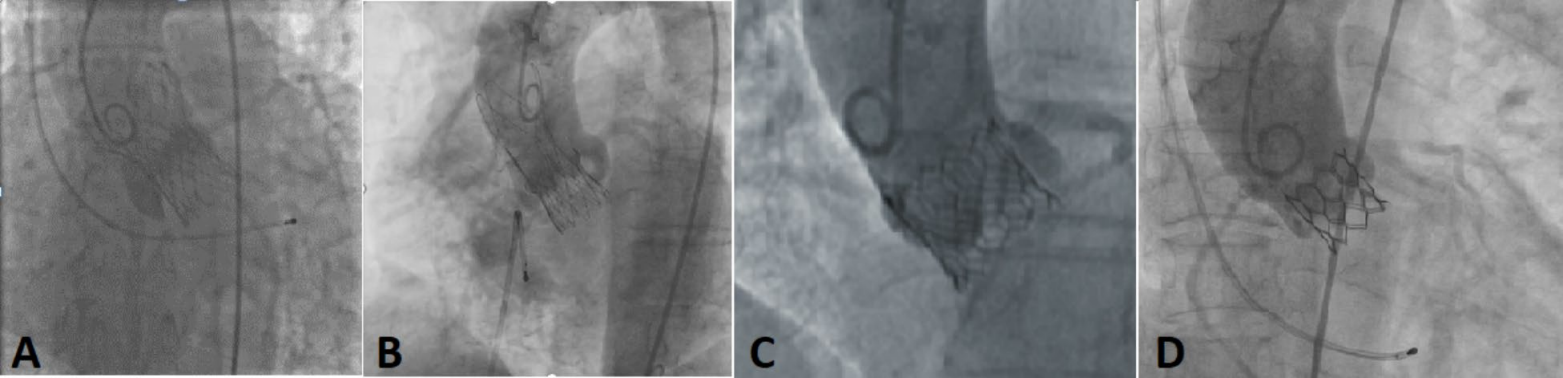
Different TAVI platforms used in rheumatic AS

Standard procedural steps for TAVI were implemented [8]. Valve options available were Evolut R™ (Medtronic, USA), Acurate Neo 2™ (Boston Scientific, USA), Sapien 3™ (Edward’s Life Sciences, USA) and MyVal™ (Meril life, India) (Fig. 2) Postimplantation balloon valvuloplasty was performed in patients with moderate AR or with a residual significant pressure gradient across the valve.

The depth of implantation was defined as the distance the between the native aortic annulus plane and the medial border of the implanted valve. The membranous septum length–implantation depth (ΔMSID) and the percentage of depth of implantation to the membranous septum (DIMS) were the two metrics used to evaluate the correlation the between the membranous septum and depth of implantation [9].

### Postprocedural outcomes

Clinical follow-up, electrocardiogram (ECG), and transthoracic echocardiography were performed post-procedure with the same variables being followed up at hospital discharge, 30 days after device implantation and 6 months. Procedural complications were defined according to the VARC-3 criteria [1].

### Statistical analysis

Statistical analysis was done by SPSS v26 (IBM Inc., Chicago, IL, USA). Shapiro–Wilk test was used to evaluate the normality of the distribution of data. Quantitative parametric variables were presented as mean and standard deviation (SD) and compared between the two groups utilizing unpaired Student’s *t* test. Qualitative variables were presented as frequency and percentage (%) and were analyzed utilizing the Chi-square test or Fisher’s exact test. A two-tailed *P* value < 0.05 was considered statistically significant.

Propensity score (PS) weighting analysis was done to adjust for measured confounders between both groups regarding the demographic data (TAVI in rheumatic vs. TAVI in degenerative AS). Patients’ weights were derived from the PS weighting methods, in which each patient’s weight is the probability of that patient being assigned to the opposite treatment group. The balance between groups after overlap weighting adjustment was shown by reporting the weighted covariate means (or proportions) for the 2 groups being compared. We performed a propensity score matching between patients with rheumatic AS and without rheumatic AS who underwent TAVI, regarding the pre-procedural data collected including demographic data, ECG, echocardiography, and CT data.

## Results

Table 1 describes the demographic data and ECG of the studied groups before and after propensity score matching. IHD, CVS and CLD were significantly lower in the rheumatic AS group than the degenerative AS group after adjustment (*P* value < 0.05).

**Table 1:** Demographic data and ECG of the studied groups before and after propensity score matching

	Before adjustment			After adjustment		
	RHD (*n* = 54)	Deg AS (*n* = 282)	*P* value	RHD (*n* = 54)	Deg AS (*n* = 54)	*P* value
Age (years)						
Mean ± SD	72.35 ± 5.86	74.46 ± 6.82	0.034*	72.35 ± 5.86	74.35 ± 7.35	0.121
Range	57–83	53–93		57–83	53–90	
Sex						
Male	16 (29.63%)	137 (48.58%)	0.01*	16 (29.63%)	21 (38.89%)	0.310
Female	38 (70.37%)	145 (51.42%)		38 (70.37%)	33 (61.11%)	
Weight (kg)						
Mean ± SD	78.72 ± 14.34	81.52 ± 15.83	0.234	78.72 ± 14.34	79.43 ± 12.48	0.823
Range	58–120	44–146		58–120	54–110	
Height (m)						
Mean ± SD	1.64 ± 0.07	1.66 ± 0.08	0.251	1.64 ± 0.07	1.66 ± 0.07	0.292
Range	1.52–1.9	1.41–1.95		1.52–1.9	1.55–1.82	
BMI (kg/m^2^)						
Mean ± SD	28.93 ± 5.23	29.45 ± 6.44	0.577	28.93 ± 5.23	29.03 ± 5.74	0.924
Range	20.1–44.08	0–54.69		20.1–44.08	15.5–52	
BSA (m^2^)						
Mean ± SD	1.86 ± 0.18	2.56 ± 10.41	0.624	1.86 ± 0.18	5.11 ± 23.7	0.317
Range	1.6–2.5	1.35–176		1.6–2.5	1.43–176	
Euro Score II						
Mean ± SD	4.59 ± 2.64	7.98 ± 8.8	0.008*	4.59 ± 2.64	5.31 ± 5.94	0.381
Range	0.91–12.4	0.84–43.84		0.91–12.4	0.84–43.84	
STS PROM						
Mean ± SD	2.78 ± 1.36	4.39 ± 3.29	0.001*	2.78 ± 1.36	3.36 ± 2.52	0.098
Range	0.8–6.9	0.02–16.98		0.8–6.9	1.1–16.98	
CABG						
Yes	4 (7.41%)	20 (7.09%)	1	4 (7.41%)	8 (14.81%)	0.359
Valve in valve						
Yes	1 (1.85%)	2 (0.71%)	0.409	1 (1.85%)	2 (3.7%)	1
Smoking						
Yes	6 (11.11%)	48 (17.02%)	0.404	6 (11.11%)	7 (12.96%)	0.252
EX	2 (3.7%)	17 (6.03%)		46 (85.19%)	47 (87.04%)	
DM						
Yes	30 (55.56%)	127 (45.04%)	0.155	30 (55.56%)	29 (53.7%)	0.847
HTN						
Yes	43 (79.63%)	210 (74.47%)	0.420	43 (79.63%)	42 (77.78%)	0.814
IHD						
Yes	15 (27.78%)	110 (39.01%)	0.117	15 (27.78%)	25 (46.3%)	0.046*
CVS						
Yes	0 (0%)	29 (10.28%)	0.007*	0 (0%)	13 (24.07%)	0.0001*
Chronic lung disease						
Yes	2 (3.7%)	32 (11.35%)	0.135	2 (3.7%)	12 (22.22%)	0.007*
CrCl (mL/min)						
Mean ± SD	61.74 ± 20.64	60.97 ± 25.09	0.833	61.74 ± 20.64	57.53 ± 21.96	0.312
Range	14–110	9–152		14–110	14–112.05	
AF						
Permanent	11 (20.37%)	33 (11.7%)	0.223	11 (20.37%)	14 (25.93%)	0.503
Paroxysmal	3 (5.56%)	17 (6.03%)		3 (5.56%)	1 (1.85%)	
BBB						
LBBB	4 (7.41%)	31 (10.99%)	0.379	4 (7.41%)	3 (5.56%)	0.471
RBBB	2 (3.7%)	28 (9.93%)		2 (3.7%)	6 (11.11%)	
IVCD	2 (3.7%)	10 (3.55%)		2 (3.77%)	1 (1.85%)	
PR						
Mean ± SD	128.15 ± 74.61	151.45 ± 61.9	0.015*	128.15 ± 74.61	142.39 ± 77.33	0.332
Range	0–240	0–320		0–240	0–240	
QRS						
Mean ± SD	95.56 ± 18.29	99.63 ± 25.03	0.256	95.56 ± 18.29	99.44 ± 23.9	0.345
Range	80–160	60–200		80–160	60–160	

*STS* *PROM* Society of Thoracic Surgeons Predicted Risk of Mortality, *BMI* Body mass index, *BSA* Body surface area

*Significant as *P* value < 0.05

Table 2 depicts the echocardiographic and CT data of the studied groups before and after propensity score matching. MPG was significantly lower in the rheumatic AS group than the degenerative AS group after adjustment (*P* value = 0.02). Ann. Diam. was significantly lower in the rheumatic AS group than the degenerative AS group after adjustment (*P* value = 0.015). Grade of Ca was significantly different between both groups after adjustment (*P* value < 0.001). Moreover, septal Ca was significantly lower in the rheumatic AS group than the degenerative AS group after adjustment (*P* value < 0.001). Calcification grading in the CT was significantly different due to the underlying different pathologies in rheumatic severe aortic stenosis versus the calcific degenerative ones.

**Table 2:** Echocardiographic and CT data of the studied groups before and after propensity score matching

	Before adjustment			After adjustment		
	RHD (*n* = 54)	Deg AS (*n* = 282)	*P* value	RHD (*n* = 54)	Deg AS (*n* = 54)	*P* value
Septal wall thickness						
Mean ± SD	12.36 ± 1.28	13.44 ± 2.06	0.001*	12.36 ± 1.28	12.64 ± 1.92	0.301
SWTi						
Mean ± SD	6.69 ± 0.9	6.7 ± 1.34	0.959	6.69 ± 0.9	7.05 ± 1.5	0.129
Posterior wall thickness						
Mean ± SD	12.26 ± 1.22	12.84 ± 1.7	0.044*	12.26 ± 1.22	12.37 ± 1.59	0.617
PWTi						
Mean ± SD	6.64 ± 0.91	6.58 ± 1.16	0.746	6.64 ± 0.91	6.74 ± 1.36	0.646
EF (%)						
Mean ± SD	57.5 ± 12.97	58.44 ± 13.46	0.635	57.5 ± 12.97	59.56 ± 13.09	0.414
LVEDD (mm)						
Mean ± SD	51.54 ± 5.23	50.98 ± 6.9	0.576	51.54 ± 5.23	51.15 ± 6.32	0.728
LVEDDi						
Mean ± SD	27.85 ± 3.46	27.11 ± 5.34	0.326	27.85 ± 3.46	26.87 ± 5.41	0.261
LVESD (mm)						
Mean ± SD	34.7 ± 6.71	34.02 ± 7.75	0.548	34.7 ± 6.71	33.87 ± 8.04	0.560
LVESDi						
Mean ± SD	18.76 ± 3.9	18.21 ± 5.01	0.444	18.76 ± 3.9	17.78 ± 5.03	0.263
PPG (mmHg)						
Mean ± SD	75.42 ± 18.01	85.57 ± 18.51	0.005*	75.42 ± 18.01	80.8 ± 19.65	0.063
MPG (mmHg)						
Mean ± SD	45.63 ± 11.86	52.43 ± 10.51	0.002*	45.63 ± 11.86	49.91 ± 12.43	0.02*
AVA (cm^2^)						
Mean ± SD	0.73 ± 0.17	0.74 ± 0.47	0.805	0.73 ± 0.17	0.77 ± 0.16	0.177
AVAi						
Mean ± SD	0.39 ± 0.1	0.4 ± 0.27	0.913	0.39 ± 0.1	0.4 ± 0.1	0.522
Aortic regurgitation						
Grade I	28 (51.85%)	161 (57.09%)	0.306	28 (51.85%)	24 (44.44%)	0.179
Grade II	22 (40.74%)	79 (28.01%)		22 (40.74%)	20 (37.04%)	
Grade III	1 (1.85%)	14 (4.96%)		1 (1.85%)	7 (12.96%)	
Grade IV	0 (0%)	3 (1.06%)		0 (0%)	0 (0%)	
RVSP (mmHg)						
Mean ± SD	44.43 ± 11.94	45.33 ± 11.94	0.612	44.43 ± 11.94	47.02 ± 12.23	0.268
Annular Diameter						
Mean ± SD	22.36 ± 2.31	23.71 ± 2.2	< 0.001*	22.36 ± 2.31	23.53 ± 2.3	0.015*
Ann. Diam. I						
Mean ± SD	10.46 ± 4.4	11.69 ± 3.8	0.036*	10.46 ± 4.4	11.45 ± 3.76	0.211
Annular Perimeter						
Mean ± SD	87.91 ± 11.99	76 ± 8.23	0.075	87.91 ± 11.99	74.84 ± 6.9	0.390
Ann. Perim. I						
Mean ± SD	46.9 ± 6.218	40.45 ± 6.39	0.089	46.9 ± 6.218	39.25 ± 6.75	0.371
Annular Area						
Mean ± SD	409.88 ± 78.42	444.26 ± 90.39	0.009*	409.78 ± 79.16	425.96 ± 75.49	0.282
Ann. Area I						
Mean ± SD	216.84 ± 51.74	235.95 ± 53.08	0.016*	216.84 ± 51.74	222.32 ± 46.53	0.564
Left main coronary						
Mean ± SD	12.11 ± 2.26	12.79 ± 2.27	0.045*	12.11 ± 2.26	12.43 ± 1.8	0.418
LMCAi						
Mean ± SD	6.55 ± 1.35	6.81 ± 1.49	0.234	6.55 ± 1.35	6.5 ± 1.31	0.841
Right coronary						
Mean ± SD	14.03 ± 3.08	14.21 ± 3.07	0.689	14.03 ± 3.08	13.44 ± 2.45	0.276
RCAi						
Mean ± SD	7.58 ± 1.76	7.57 ± 1.88	0.983	7.58 ± 1.76	7.04 ± 1.55	0.093
Membranous septum						
Mean ± SD	9.07 ± 1.93	8.14 ± 2.29	0.025*	9.07 ± 1.93	8.9 ± 2.25	0.620
MSi						
Mean ± SD	4.9 ± 1.15	4.24 ± 1.34	0.007*	4.9 ± 1.15	4.69 ± 1.4	0.299
Grade of calcification						
Grade I	23 (42.59%)	41 (14.54%)	< 0.001*	23 (42.59%)	9 (16.67%)	< 0.001*
Grade II	19 (35.19%)	125 (44.33%)		19 (35.19%)	16 (29.63%)	
Grade III	6 (11.11%)	71 (25.18%)		6 (11.11%)	14 (25.93%)	
Grade IV	1 (1.85%)	45 (15.96%)		1 (1.85%)	15 (27.78%)	
Septal calcification						
Yes	1 (1.85%)	32 (11.35%)	0.041*	1 (1.85%)	13 (24.07%)	< 0.001*
Aortic angulation						
Mean ± SD	44.22 ± 7.68	46.26 ± 9.92	0.162	44.22 ± 7.68	42.75 ± 8.23	0.510

*Significant as *P* value < 0.05

Table 3 describes comparison between procedural details in both groups before and after propensity score matching. Pre-dilatation, depth of implantation (DI), DIi and DIMS were significantly higher in the rheumatic AS group than the degenerative AS group after adjustment (*P* value < 0.05). ΔMSID was significantly lower in the rheumatic AS group than the degenerative AS group after adjustment (*P* value < 0.05).

**Table 3:** Comparison between procedural details in both groups before and after propensity score matching

Procedure	RHD (54)	Deg (282) before adjustment	*P* value	Deg (54) after adjustment	*P* value
Valve type					
Sapien XT	0 (0%)	11 (3.9%)		0 (0%)	
Corevalve	0 (0%)	3 (1.06%)		0 (0%)	
Evolut R	43 (79.63%)	235 (83.33%)		42 (77.78%)	
Sapien 3	1 (1.85%)	5 (1.77%)		2 (3.7%)	
Acurate Neo 2	9 (16.67%)	24 (8.51%)		10 (18.52%)	
MyVal	1 (1.85%)	2 (0.71%)		0 (0%)	
Evolut Pro	0 (0%)	2 (0.71%)		0 (0%)	
Valve size Mean ± SD	28.93 ± 3.42	29.55 ± 2.92		28.11 ± 2.23	
Depth of implantation (DI) Mean ± SD	4.41 ± 2.73	3.62 ± 2.12	0.048	3.44 ± 1.34	0.021*
DI index mean ± SD	2.34 ± 1.43	1.93 ± 1.13	0.051	1.85 ± 0.71	0.024*
Pre-dilatation	34 (62.96%)	120 (42.55%)	0.006*	20 (37.04%)	0.007*
Post-dilatation	9 (16.7%)	57 (20.21%)	0.548	12 (22.22%)	0.465
DIMS mean ± SD	58.95 ± 37.76	43.59 ± 27.7	0.006	39.66 ± 17.58	< 0.001*
MSID mean ± SD	3.48 ± 3.43	5.2 ± 3.12	0.001	5.63 ± 2.42	< 0.001*
Approach					
Percutaneous Surgical	17 (31.5%)	92 (32.62%)		15 (27.78%)	
	37 (68.5%)	190 (67.38%)		39 (72.22%)	
Access site					
Femoral Carotid 3rd part axillary 1st part axillary	53 (98.1%)	279 (98.94%)		53 (98.15%)	
	0 (0%)	2 (0.71%)		1 (1.85%)	
	0 (0%)	1 (0.35%)		0 (0%)	
	1 (1.9%)	0 (0%)		0 (0%)	

Figures presented as mean ± SD and range; number (percentage)

Table 4 illustrates the primary outcomes according to VARC-3 and further secondary outcomes of the procedure. The outcomes were categorized according to VARC-3 criteria. One patient experienced cardiac structural complication of valve embolization. A total of 3.7% of the patients had major vascular complications requiring vascular surgery intervention. Trace/mild cases of PVL were evident in 25.9% of the patients. One patient was presented with acute kidney injury during the 6-month follow-up. Moreover, one patient experienced neurological insult 2-month post-procedure. All-cause death within 30 days accounted for 3.7% of the cases. Secondary outcomes were also analyzed. Overall, 32.5% of the total conduction disturbances were further classified into transient LBBB 20.4%, transient atrio-ventricular block (AVB) (9.3%) and permanent left bundle branch block (LBBB) (7.4%), and permanent AVB (3.7%). Notably the patients who developed transient AVB experienced transient LBBB recovered back to narrow complex sinus rhythm with one to one conduction within 48 h of continuous monitoring. Two patients died early within 30 days; thus, 52 patients were followed up at 6 months. Total conduction disturbances are closely related to pre-procedural echocardiographic septal wall thickness with a *P* value of 0.041. However, they were not related to any pre-procedural ECG criteria, other echocardiographic parameters, or CT data. Total conduction disturbances are related to procedural ratio between the depth of implantation and the membranous septum with a *P* value of 0.034 and are also related to the difference between the membranous septum and the depth of implantation with a *P* value of 0.034. However, they were not related to the type of the valve, valve size, nor balloon dilatation. Trace/mild PVL was not associated with the type, nor size of the trans-catheter heart valve. In addition, it is not related to the depth of implantation, balloon dilatation, DIMS, MSID, nor approach.

**Table 4:** Primary outcomes according to VARC- 3 and further secondary outcomes in the rheumatic group

1ry outcomes		Within 30 days	Within 6 months
New conduction disturbances		17 (31.4%)	17 (31.4%)
Permanent pacemaker implantation		2 (3.7%)	2 (3.7%)
Bio-prosthetic valve dysfunction (mild PVL)		14 (25.9%)	14 (25.9%)
Cardiac structural complications (valve embolization)		1 (1.85%)	1 (1.85%)
Major vascular complications		2 (3.7%)	2 (3.7%)
Bleeding and transfusions		1 (1.85%)	1 (1.85%)
Neurological manifestations		1 (1.85%)	2 (3.7%)
Acute kidney injury		0 (0%)	1 (1.85%)
Myocardial infarction		1 (1.85%)	1 (1.85%)
Mortality		2 (3.7%)	2 (3.7%)
2ry outcomes No. = 54			
Total Conduction Disturbances		17 (31.5%)	
Left bundle branch block			
Transient			
Permanent			
Atrio-ventricular block (AVB)		11 (20.4%)	
		4 (7.4%)	
Transient			
Permanent		5 (9.3%)	
		2 (3.7%)	
QRS widening		13 (24.5%)	
PR Prolongation		7 (13.2%)	
New onset atrial fibrillation		4 (7.4%)	

**Fig. 3:**
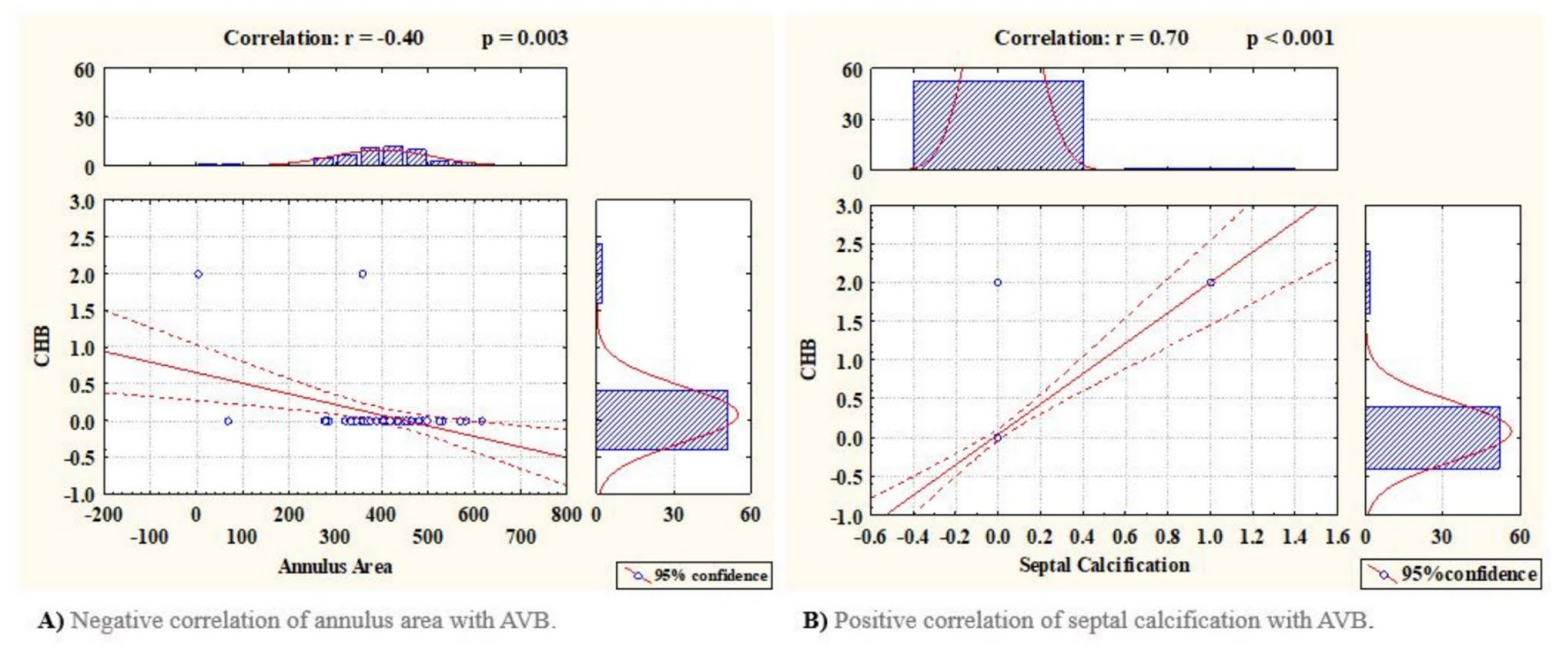
Correlations between the annular area and septal calcification with AVB

Figures presented as number (percentage)

a *P* value of 0.33 (Fig. 3).

Table 5 demonstrates comparison between post-procedural outcomes in both groups before and after propensity score matching. Conduction disturbance, LBBB, CHB and Transient conduction disturbance were insignificantly different between both groups after adjustment. QRS widening, PR Prolongation, new-onset AF, major Vasc. Comp., PVL, FUP 1 months and FUP 6 months were insignificantly different between both groups after adjustment. Death in 30 days was insignificantly different between both groups after adjustment.

**Table 5:** Comparison between post-procedural outcomes in both groups before and after propensity score matching

	Before adjustment			After adjustment		
	RHD (*n* = 54)	Deg AS (*n* = 282)	*P* value	RHD (*n* = 54)	Deg AS (*n* = 54)	*P* value
Conduction disturbance						
Transient	17 (31.48%)	92 (32.62%)	0.869	17 (31.48%)	24 (44.44%)	0.165
LBBB						
Grade I	11 (20.37%)	40 (14.18%)	0.418	11 (20.37%)	15 (27.78%)	0.659
Grade II	4 (7.41%)	31 (10.99%)		4 (7.41%)	4 (7.41%)	
CHB						
Grade I	0 (0%)	15 (5.32%)	0.119	0 (0%)	3 (5.56%)	0.185
Grade II	2 (3.7%)	4 (1.42%)		2 (3.7%)	1 (1.85%)	
Transient conduction disturbance						
Yes	5 (9.26%)	37 (13.12%)	0.431	5 (9.26%)	5 (9.26%)	1
QRS widening						
Yes	13 (24.07%)	53 (18.79%)	0.371	13 (24.07%)	17 (31.48%)	0.390
PR Prolongation						
Yes	7 (12.96%)	26 (9.22%)	0.397	7 (12.96%)	8 (14.81%)	0.780
New onset AF						
Yes	4 (7.41%)	5 (1.77%)	0.133	4 (7.41%)	1 (1.85%)	0.363
Major Vasc. Comp						
Yes	2 (3.7%)	16 (5.67%)	0.133	2 (3.7%)	3 (5.56%)	1
PVL						
Mild	14 (25.93%)	89 (31.56%)	0.411	14 (25.93%)	22 (40.74%)	0.102
FUP 1 months						
Good clinical condition	50 (96.15%)	264 (97.42%)	0.386	50 (96.15%)	50 (98.04%)	1
Co-morbidity	2 (3.85%)	4 (1.48%)		2 (3.85%)	1 (1.96%)	
Death	0 (0%)	3 (1.11%)		0 (0%)	0 (0%)	
FUP 6 months						
Good clinical condition	50 (96.15%)	261 (97.39%)	0.239	50 (96.15%)	50 (100%)	0.495
Co-morbidity	2 (3.85%)	3 (1.12%)		2 (3.85%)	0 (0%)	
Death	0 (0%)	4 (1.49%)		0 (0%)	0 (0%)	

*Significant as *P* value < 0.05

(small annuli representing 430 mm^2^ or less) (*r* = − 0.40 and *P* = 0.003) (*r* = 0.70 and *P* < 0.001).

## Discussion

A new era in the management of valvular heart disease has been brought about implementing TAVI. The majority of research has gone into treating calcific degenerative disease in populations; however, rheumatic heart disease (RHD) causing severe aortic stenosis is still a condition that is not well understood [10]. In addition to determining the post-procedural outcomes of TAVI in patients with rheumatic aortic stenosis in respect to VARC-3 criteria [1], our study is the first to our knowledge to characterize the correlation between outcomes and pre-procedural and procedural data.

Evidence of performing routine pre-dilatation to rheumatic severe aortic stenosis is lacking. Initially, our early interventions did not include routine pre-dilatation unless the case involved very severe aortic stenosis or during implantation of the Acurate Neo 2 THV platform. Consequently, experiencing valve embolization in one of our cases using Evolut R THV platform after implantation due to evident restrictions around the valve by fluoroscopy gave us an insight to implement routine pre-dilatation in the remaining cases. Our explanation is that the fibrosis and commissural fusion at the valve act as an elastic band imposing high external pressure on the valve causing embolization. Thus, pre-TAVI balloon commissurotomy becomes mandatory to decrease the pressure imposed on the implanted valve, according to our experience.

Surgery is the recommended course of action for patients with severe stenosis brought on by a rheumatic aortic valve. Mentias et al. [11] gathered data on the surgical outcomes of rheumatic aortic stenosis and reported that in the propensity matched group, the incidence of acute renal injury following surgery was 22.3%, the rate of permanent pacemaker insertion was 7.2%, and the 30-day mortality rate was 3.2%. Our study revealed that the incidence of acute renal injury was 1.9%, the mortality rate in 30 days was 3.7%, and the incidence of permanent pacemaker implantation was 3.7%. These results demonstrate that in patients with severe rheumatic aortic stenosis, TAVI is non-inferior to SAVR. However, multiple factors were confounding including our small sample size, and different ages along with the mortality risk of the population treated in the Mentias et al. [11] group (higher age and mortality risk).

Propensity score matched, our rheumatic severe aortic stenosis group post-procedural data were comparable with that of the degenerative aortic stenosis group. This gives us insights and support regarding the feasibility and efficacy of implementing trans-catheter aortic valve implantation in rheumatic severe aortic stenosis patients. The similar outcomes with TAVI for rheumatic versus non-rheumatic AS patients confirm the safety and the feasibility of TAVI in rheumatic AS.

**Fig. 4:**
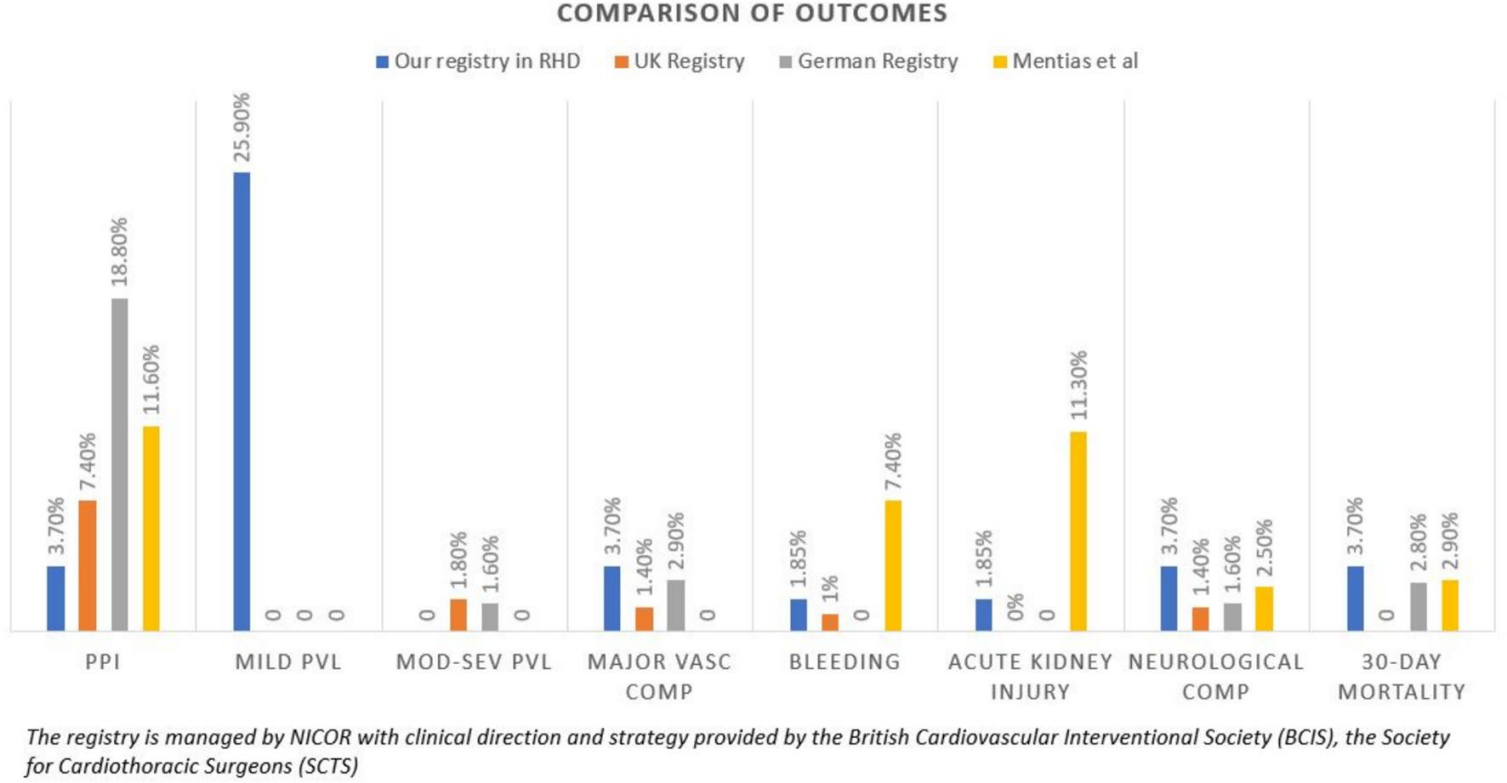
UK, German, and USA TAVI registries describing the outcomes in non-rheumatic AS in comparison to our registry

Going with our results, 30-day mortality rate detected by Mentias et al. [11] of 3.6% in the rheumatic group who underwent TAVI was concordant to our findings. Regarding the recent UK [overseen by NICOR, using a clinical plan and guidance from the British Cardiovascular Interventional Society (BCIS), and the Society for Cardiothoracic Surgeons (SCTS)], German [12] and Mentias et al. [11] registries of TAVI in non-rheumatic AS patients, the outcomes revealed by our study of rheumatic aortic stenosis are comparable with respect to all-cause death (mortality), yet the difference in the sample size is a drawback. These findings suggest that TAVI is feasible and safe for treating rheumatic AS. In fact, more data are yet to be collected to compare the behavior of the implanted THV in rheumatic aortic valves properly (Fig. 4).

## Conclusion

Our study is the largest cohort to date with specified clinical characteristics, ECG criteria, echocardiographic parameters, and CT data to highlight the suitability of TAVI in patients with rheumatic AS. Although it is anticipated that the technical success of TAVI in RHD patients is lower due to the aforementioned anatomical differences, the feasibility and effectiveness of TAVI in rheumatic patients is encouraging. Additionally, the safety of TAVI in these RHD patients is confirmed by the comparable results with TAVI for rheumatic versus degenerative aortic stenosis as represented by our propensity scored matched analysis of our two groups of patients. For patients with rheumatic AS, TAVI actually provides a viable and reasonable choice compared with the results of the procedure in cases of degenerative aortic stenosis.

## Limitations

This study has the usual drawbacks of being a single-center observational nonrandomized investigation chiefly because of the small study population. Furthermore, a comparison with patients with rheumatic aortic stenosis who undergo surgical aortic valve replacement (SAVR) is necessary to assess the viability of TAVI in treating rheumatic aortic stenosis. In fact, longer follow-up is needed to identify long-term issues.

## Recommendations

A larger prospective sample size is needed to obtain a broader understanding of the THV behavior in rheumatic aortic valves. Further randomized controlled trials are needed to compare TAVI in rheumatic severe aortic stenosis patients and SAVR in rheumatic severe aortic stenosis patients. Validation of echocardiographic and CT phenotyping of rheumatic aortic valves is through histopathological examination.

## Data Availability

Data is available upon request from the corresponding author.
